# Different Roles of Auxins in Somatic Embryogenesis Efficiency in Two *Picea* Species

**DOI:** 10.3390/ijms21093394

**Published:** 2020-05-11

**Authors:** Teresa Hazubska-Przybył, Ewelina Ratajczak, Agata Obarska, Emilia Pers-Kamczyc

**Affiliations:** Institute of Dendrology, Polish Academy of Sciences, 62-035 Kórnik, Poland; eratajcz@man.poznan.pl (E.R.); aobarska@man.poznan.pl (A.O.); epk@man.poznan.pl (E.P.-K.)

**Keywords:** picloram, somatic embryos, spruce, hydrogen peroxide, peroxidases

## Abstract

The effects of auxins 2,4-D (2,4-dichlorophenoxyacetic acid), NAA (1-naphthaleneacetic acid) or picloram (4-amino-3,5,6-trichloropicolinic acid; 9 µM) and cytokinin BA (benzyloadenine; 4.5 µM) applied in the early stages of somatic embryogenesis (SE) on specific stages of SE in *Picea abies* and *P. omorika* were investigated. The highest SE initiation frequency was obtained after 2,4-D application in *P. omorika* (22.00%) and picloram application in *P. abies* (10.48%). NAA treatment significantly promoted embryogenic tissue (ET) proliferation in *P. abies*, while 2,4-D treatment reduced it. This reduction was related to the oxidative stress level, which was lower with the presence of NAA in the proliferation medium and higher with the presence of 2,4-D. The reduced oxidative stress level after NAA treatment suggests that hydrogen peroxide (H_2_O_2_) acts as a signalling molecule and promotes ET proliferation. NAA and picloram in the proliferation medium decreased the further production and maturation of *P. omorika* somatic embryos compared with that under 2,4-D. The quality of the germinated *P. abies* embryos and their development into plantlets depended on the auxin type and were the highest in NAA-originated embryos. These results show that different auxin types can generate different physiological responses in plant materials during SE in both spruce species.

## 1. Introduction

Somatic embryogenesis (SE) is one of the most efficient in vitro regeneration systems for some coniferous tree species [1]. Together with cryopreservation and genetic transformation techniques, it represents a modern biotechnological tool for the mass vegetative propagation of economically and ecologically important trees. The process of SE in conifers is very complex and includes many steps [2]. In the SE process, embryos develop from somatic cells without fertilization. The obtained embryos (so-called somatic embryos) have the same genotype as the somatic cells, and they strongly resemble zygotic embryos in seeds. Properly developed somatic embryos have a typical bipolar structure with a shoot and root apices and can germinate and grow into somatic seedlings.

Plant growth regulators (PGRs) fundamentally influence the course of the particular developmental steps of SE in plants [3]. Many studies have investigated the phytohormonal regulation of embryo development in conifer species, but the effects of exogenously applied PGRs on changes in cellular metabolism are largely unknown. The role of PGRs in this process has been examined widely based on ‘one-factor-at-a-time‘ and ‘trial-and-error’ techniques. However, the hormonal requirements have been optimized only for certain cultivars or genotypes [3]. To improve the efficiency of the SE technique, it is necessary to eliminate the multiple problems that persist for many tree species. Most of these problems are connected with the type and concentration of PGRs added to the induction and proliferation media, especially with synthetic auxins, which do not have exactly the same mechanism of action as natural auxin, IAA (indole-3-acetic acid) [4].

In most conifer species, particularly in *Picea*, *Pinus,* and *Larix* genus, PGRs in the auxin and cytokinin groups are some of the main factors that affect the induction of embryogenic cultures [5]. Thus, they determine the acquisition of totipotency by explant cells [6] and consequently induce the SE process. They are involved in the regulation of cell division and differentiation processes in plant cells [7], leading to the formation of somatic embryos at an early stage of development.

2,4-D (2,4-dichlorophenoxyacetic acid) is the most widely studied synthetic auxin and is applied in different plant embryogenic systems [8], including conifer SE. Other synthetic auxins, such as NAA (1-naphthaleneacetic acid) and picloram (4-amino-3,5,6-trichloropicolinic acid), are believed to be less effective in coniferous species [9] although they have often been effective in other plants [10,11]. Nevertheless, positive results for these two auxins in combination with benzyloadenine (BA) have also been reported during the induction of SE and proliferation of embryogenic tissues (ETs) in *Picea abies* and *P. glauca* [12,13] and in other tree species (*P. omorika*, *P. koraiensis*, *Larix kaempferi*, and *Cordyline australis*) [14,15,16,17]. Exogenously applied auxins are known to regulate somatic embryo development by changing the level of endogenous auxins such as IAA [3]. Therefore, the role of these synthetic auxins in the promotion of SE in some coniferous species should also be deeply studied. This is especially important in the case of recalcitrant species for which it is difficult to establish efficient multiplication protocols using synthetic auxins.

Coniferous ETs are usually maintained long-term on media supplemented with both auxin and cytokinin [18,19,20]. However, in vitro tissue subcultures often lose their embryogenic potential or transition their embryogenic tissue into nonembryogenic callus. This limits the use of the SE technique in clonal forestry [21] and is one of the unresolved problems that may be connected with the type and concentration of PGRs used in the proliferation medium. To overcome this problem, knowledge about the long-term effects of various auxins/cytokinin combinations added to in vitro maintained tissue media and their metabolism is needed.

The exposure of plant cells to in vitro conditions is connected with various types of stress, including oxidative stress, the wounding of explant tissue, the concentration of macro-and micronutrients in the medium, the light conditions and the type of PGRs added to the medium [22]. It is believed that oxidative stress can serve as a modulator of SE in plants through the induction of autonomous cell division [23]. Reactive oxygen species (ROS) are also involved in the growth, differentiation and development of plant cells, including the process of in vitro morphogenesis [24]. At low concentrations, ROS (H_2_O_2_ especially) act as signal molecules that initiate metabolic changes or stimulate plant regeneration [25,26]. However, the knowledge of how ROS regulate plant growth and development under stress conditions (including in vitro cultures) and how they interact with plant hormones has not been sufficiently explored. According to Zavattieri et al. [27], oxidative stress and the SE process may be linked. Moreover, significant amounts of stress induced by both PGRs and ROS, especially H_2_O_2_, are necessary to trigger SE as well as to promote the regeneration of somatic embryos in tree species [28]. Hydrogen peroxide (H_2_O_2_) interacts with thiol-containing proteins and activates different signalling pathways as well as transcription factors, which in turn regulate gene expression and cell-cycle processes [29] and are involved in the regulation of root development [30]. Peroxidases (EC 1.11.1.7) are one of the groups of enzymes whose activity changes at various stages of somatic embryogenesis in plants [31]. The main functions of these enzymes are to protect cells from oxidation by peroxides generated during biochemical processes and to participate in plant cell development [32], as well as in somatic embryos of coniferous tree species [33,34].

The effects of various synthetic auxins on the physiological responses of plant materials during the induction and development of conifer somatic embryos have not been compared, therefore, this study aimed to: (1) to increase our knowledge of whether the type of synthetic auxin used in the early stages of somatic embryogenesis in *P. abies* and *P. omorika* determine the efficiency of induction, proliferation, and maturation processes and the further growth and development of the somatic embryos into plants and (2) to verify whether the level of H_2_O_2_ accumulation in embryogenic tissues during their maintenance in the presence of individual auxins caused oxidative stress or was associated with the signal function of this molecule.

## 2. Results

### 2.1. Initiation and Maintenance of Embryogenic Cultures

**Figure 1:**
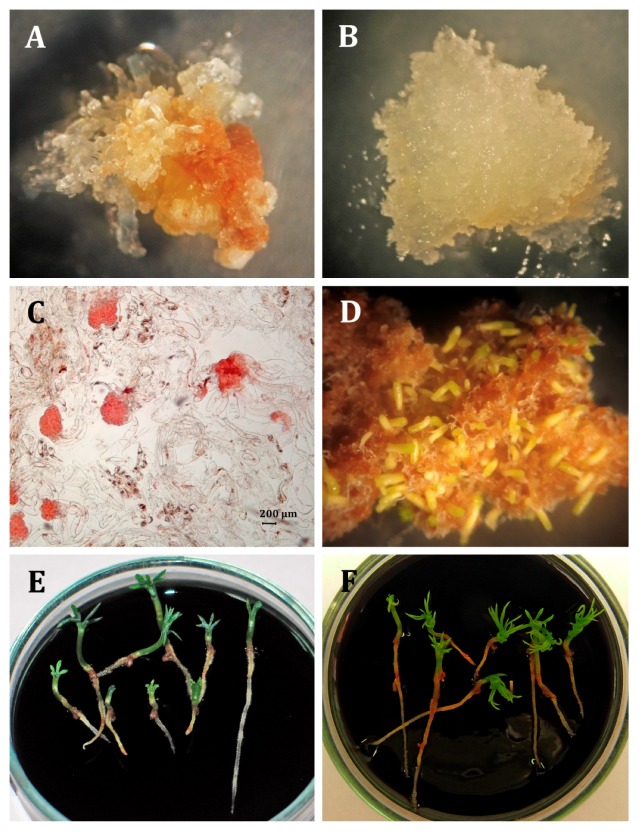
Somatic embryogenesis in *P. abies*. (**A**) Embryogenic masses developed on the explant. (**B**) Embryogenic cultures growing on the proliferation medium. (**C**) Proembryogenic structures (proembryos) present in the embryogenic tissues. (**D**) Somatic embryos going through the maturation process. (**E**) Germinated somatic embryos. (**F**) Somatic seedlings with developed first needles suitable for acclimatization.

**Figure 2:**
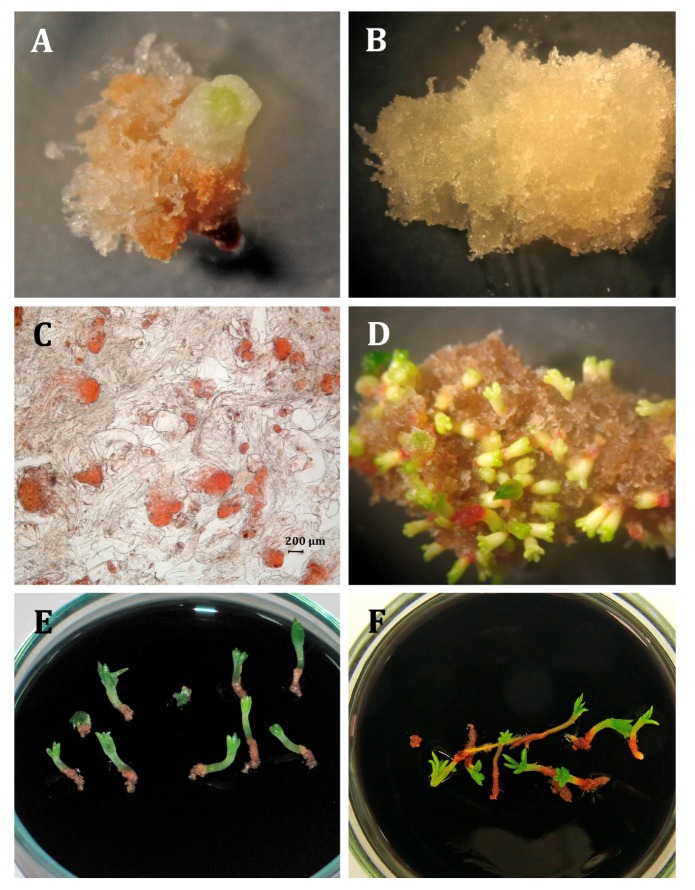
Somatic embryogenesis in *P. omorika*. (**A**) Embryogenic masses developed on the explant. (**B**) Embryogenic cultures growing on the proliferation medium. (**C**) Proembryogenic structures (proembryos) present in the embryogenic tissues after staining with acetocarmine. (**D**) Somatic embryos going through the maturation process. (**E**) Germinated somatic embryos. (**F**) Somatic seedlings with developed first needles suitable for acclimatization.

Embryonic tissue initiation from mature zygotic embryos was obtained for both spruce species regardless of the auxin used (Table 1, Figure 1A,B and Figure 2A,B), and the auxin used had no significant effect on ET initiation in either species (*P. abies*: P_χ2 = 1.8114, DF = 2_ < 0.05 and *P. omorika* P_χ2 = 4.7474, DF = 2_ < 0.05). The highest percentage of explants that produced embryogenic masses was observed in *Picea abies* on the medium supplemented with picloram (10.48%, 24/229), whereas in *P. omorika*, it was observed on the medium with 2,4-D (22.00%, 55/250). On the other hand, the lowest percentage of ET initiation was observed on the medium supplemented with 2,4-D (7.33%, 17/232) in *P. abies* and on the medium supplemented with picloram (15.15%, 40/264) in *P. omorika*.

In both species, most of the initiated ET lines (*P. abies*: from 71% to 84%; *P. omorika*: from 76% to 87%) lost the ability to proliferate during the first ten passages (each after 10–11 days) of incubation on the maintenance media regardless of the auxin treatment (Table 2). Auxins had no effect on the percentage of lines characterized by the ability for in vitro growth and proliferation for more than a year (40 passages) in both the *P*. *abies* (P_χ2 = 0.3344, DF = 2_ < 0.05) and *P. omorika* ET lines (P_χ2 = 1.8979, DF = 2_ < 0.05). However, in *P. abies,* up to 30% (2,4-D line), and in *P*. *omorika*, up to 17% (NAA line) of ET lines proliferated for over a year (Table 2).

### 2.2. Effects of Auxin Treatment on the Physiological Condition of the ET Lines and the Levels of Oxidative Stress and Guaiacol Peroxidase (POX) Activity

There was a significant effect of the auxin used on the growth intensity of the ET lines of *P. abies* [F(2,10) = 3.88, *p* = 0.05, Table 3] but not on the growth intensity of the ET lines of *P*. *omorika* [F(2,15) = 1.25, *p* = 0.32]. Post hoc comparisons using the Tukey HSD test indicated that the most intensive growth (1.06 g) was obtained in the NAA ET lines of *P. abies*; the growth of these lines was significantly different from that of 2,4-D ET lines (0.58 g) but not from that of picloram ET lines (0.78 g). All tested *P. omorika* ET lines had a similar growth intensity of approximately 1.00 g.

The production of reactive oxygen species and the level of POX activity were related to the auxin used in *P. abies* but not in *P. omorika* (Table 3). In *P. abies*, the highest level of H_2_O_2_ was observed in the 2,4-D ET lines, which was significantly different from the level of H_2_O_2_ in the picloram lines but not from that in the NAA ET lines [F(2,36) = 3.9813; *p* = 0.0274]. Additionally, the highest level of POX activity was observed in the picloram ET lines, and the lowest was observed in the NAA ET lines [F(2,36) = 4.9166; *p* = 0.0129]. In *P. omorika*, the POX activity [F(2,45) = 0.3043; *p* = 0.7392] and the level of H_2_O_2_ [F(2,45) = 3.1766; *p* = 0.0512] were similar regardless of the auxin used.

### 2.3. Somatic Embryo Production

**Figure 3:**
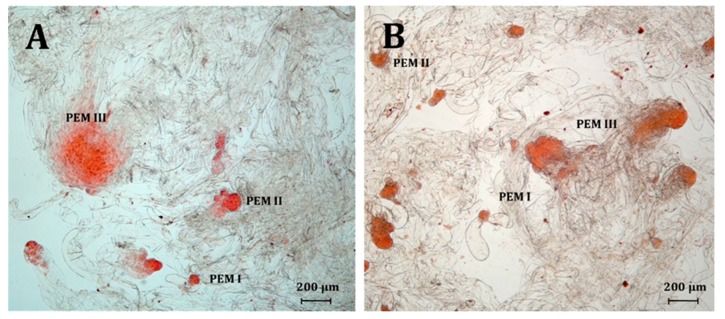
Morphology of various embryogenic structures in: (**A**) *P. abies* and (**B**) *P. omorika*. proembryonic structures (PEM) I–III – embryogenic structures type I–III.

In both species, the mean number of proembryos (Figure 3) was affected by the auxin used [*P. abies* (q = 2.36199, *p* < 0.05); *P. omorika* (q = 2.35946, *p* < 0.05)]. In *P. abies*, the mean number of proembryos observed in the NAA ET lines was significantly higher (19.02 ± 1.8 a) than that in the picloram (13.73 ± 0.9 b) and 2,4-D ET lines (9.77 ± 0.9 c). Similarly, in *P. omorika*, the NAA ET lines produced a significantly higher number of proembryos (44.95 ± 2.3 a) than the 2,4-D (35.06 ± 2.1 b) and picloram (28.09 ± 2.1 b) ET lines.

Furthermore, the percentage of proembryonic structures in the ET lines was influenced by the auxin used (Table 4). In total, 2629 proembryonic structures were obtained in *P. abies*, and more than 80% of them were classified as proembryonic structures (PEM) II or PEM III. Although more proembryonic structures (8677) were observed in *P. omorika* than in *P. abies*, fewer of them were classified as PEM II or PEM III (74.69%, 6481/8677). The growth of proembryogenic structures was affected by the auxin used. The size of the embryogenic region of PEM III structures in *P. abies* obtained from the 2,4-D (212.63 ± 6.2 µm) and picloram (205.88 ± 5.9 µm) ET lines was larger than that from the NAA ET lines (179.30 ± 7.6 µm) [F(2177) = 6.1660 *p* = 0.0026]. In *P*. *omorika*, the effect of the auxin used on the size of the embryogenic region was also observed. The mean size of the embryogenic region of PEM III structures obtained from the picloram ET lines was larger (94.47 ± 0.8 a) than those of PEM III structures of the other ET lines [F(2224) = 5.5196, *p* = 0.0046] (Table 4).

**Figure 4:**
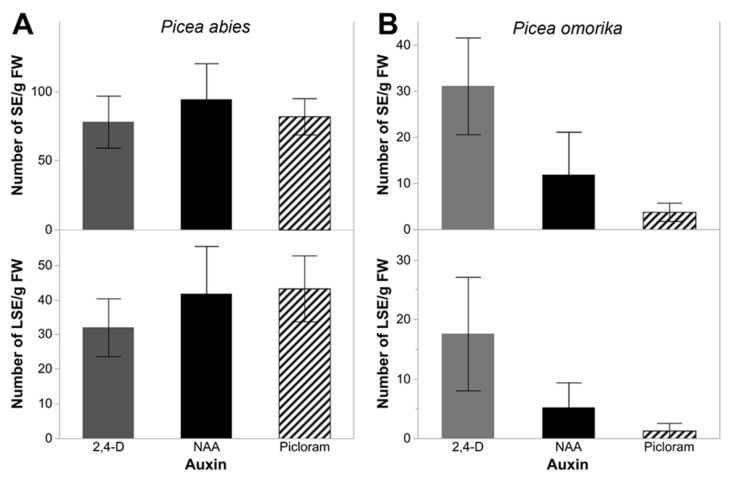
The mean number of somatic embryos and the mean number of cotyledonary stage embryos per gram of fresh weight of the tissue produced by ET lines of *P. abies* (**A**) and *P. omorika* (**B**) in relationship to the PGR type used during the induction of the ET lines. Data are means with standard errors from 5 replicates from each tested line.

ETs of both spruce species were able to regenerate somatic embryos (SESs) (Figure 1C,1D, 2C,2D) regardless of the type of synthetic auxin used, however, the embryogenic potential was lower for *P. omorika* tissues when a similar number of ET lines was tested for each species. When only ET lines that produced SESs were analysed, all but one ET line produced embryos at the cotylenodonary stage in *P. abies* (11/12), however, only half of ET lines produced cotylenodonary-stage SESs in *P. omorika* (6/12). In total, *P. abies* ET lines produced 235 SESs per gram, and 109 of them reached the cotyledonary stage, whereas *P. omorika* ET lines produced 39 SESs per gram, and only 19 of them reached the cotyledonary stage. Although auxin did not impact the number of SESs produced or the number of SESs at the cotyledonary stage in either species (*p* > 0.05) (Figure 4), a decrease in the mean number of somatic embryos was observed in the NAA and picloram ET lines compared to that in the 2,4-D line in *P. omorika*.

**Figure 5:**
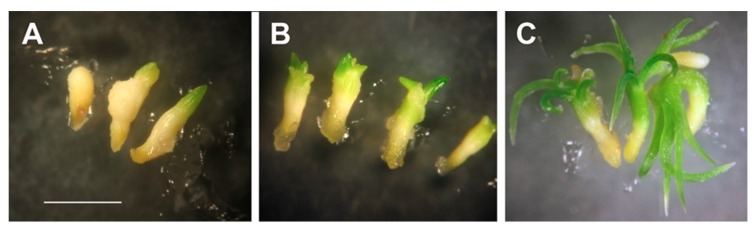
Morphological deformations of *P. abies* somatic embryos (as an example). (**A,B**) Embryos with swollen hypocotyl and/or lacking some cotyledons and (**C**) precociously germinated embryos. Bar = 1 mm.

In both *Picea* spp., the obtained cotyledonary somatic embryos were properly developed, regardless of the auxin used. Only a few embryos were characterized by morphological deformations such as a swollen hypocotyl or the lack of some cotyledons or by precocious germination (Figure 5).

### 2.4. Germination and Acclimatization

The germination of the somatic embryos (Figure 1E and Figure 2E) was influenced by auxin used in both spruce species (Table 5). In *P. abies*, SESs developed from the 2,4-D ET line had the longest hypocotyl (9.91 mm) of all lines in the first phase of germination (the first two weeks, in darkness), whereas in the second phase of germination (two weeks of SESs growth in the presence of light), SESs from the 2,4-D (11.08 mm) and picloram (10.37 mm) lines had longer hypocotyls than the SESs from the other ET lines (Table 5). In both phases of germination, the SESs from the NAA ET lines had the shortest hypocotyls. Somatic embryos from the 2,4-D (2.76 mm) and picloram (2.71 mm) lines had the longest radicle lengths in the dark, whereas the opposite trend was observed in the light, i.e., the SESs from the NAA lines (5.32 mm) had the longest radicles. The ratio of the hypocotyl length to the radical length was significantly the lowest in the germinated SESs obtained from the NAA lines (1.51, *p* < 0.05). This means that the level of synchronization of the hypocotyl to radicle development was the best in these lines. Finally, in *P. abies*, 327 germinated embryos characterized by a hypocotyl length over 10 mm and a radicle length over 5 mm (somatic seedlings) were obtained and classified as suitable for acclimatization (Figure 1F). Somatic seedlings accounted for 29.22% (327/1119) of all somatic embryos germinated in vitro. Statistical analysis indicated that the auxin treatment affected the percentage of *P. abies* somatic seedlings suitable for acclimatization (P_χ2 = 50.5011, DF = 2_ < 0.0001). The highest percentage of seedlings suitable for acclimatization was obtained in the NAA ET line (41.55%, 123/296), and the lowest percentage was obtained in the 2,4-D line (13.25%, 31/234, Table 6).

In *P. omorika*, the SESs developed the longest hypocotyls when the ETs were maintained on the medium supplemented with NAA in the early stage (4.88 mm in darkness and 5.98 mm in light conditions; Table 5). Moreover, somatic embryos in the NAA ET line had the longest hypocotyl and the shortest radicle regardless of the germination conditions, and those features were significantly different from those of the SESs in the 2,4-D ET lines but not in the picloram ET lines (*p* < 0.05). The level of synchronization of hypocotyl development to radicle development in the somatic embryos was the highest for the picloram ET line (1.78) and the lowest for the NAA (4.41) ET line (*p*_α = 0.05, q = 2.35905_ < 0.05).

Most of the germinated SESs produced only thickened brown radicles that showed weak growth, regardless of the auxin used. In many cases, no white radicles with root hairs were observed; therefore, such germinated embryos were not selected for acclimatization (Figure 2F). Only 12 properly germinated somatic embryos of *P. omorika* were obtained and classified as suitable for acclimatization in soil (Table 6).

## 3. Discussion

This is the first report in which the effects of three synthetic auxins (2,4-D, NAA and picloram) used at the early stages of somatic embryogenesis on the whole somatic embryogenesis process in two *Picea* species have been analysed. Obtained results showed that regardless of the type of auxin used, the explants (mature zygotic embryos) formed proembryogenic masses with similar frequency. They are in line with earlier studies where these auxins were used for the induction of somatic embryogenesis [14,33].

### 3.1. Initiation and Maintenance of Embryogenic Cultures

We demonstrated that mature zygotic embryos of *P. abies* and *P. omorika* are sensitive not only to the frequently used 2,4-D auxin but also to NAA and picloram. Moreover, those rarely used auxins can be used to obtain tissues that can multiply and produce properly developed somatic embryos. In the current study, *P. abies* explants showed a lower SE induction frequency (up to 10.48%) than *Picea omorika* explants (22.00%), and the best results were observed when BA, together with picloram or 2,4-D was applied to the induction medium, respectively. Our observations confirm that plant growth regulators (mainly auxins and cytokinins) play a significant role in inducing SE processes in many plants, including conifers [35].

Auxins are the key plant growth regulators that control somatic embryogenesis, both when they are exogenously used in culture media and when they are active at the cellular level [3,35,36]. According to Kawahara and Komamine [37], exogenous auxins are involved in gene expression at the early stages of SE. The exogenously applied synthetic auxin 2,4-D is commonly used to trigger the SE process from various explant types in many plant species, including coniferous trees [8]. Studies have shown that under the influence of 2,4-D, the level of endogenous auxin increases, which leads to an increase in cell division and the establishment of a hormonal gradient that is optimal for the induction of embryogenesis from the somatic cells of treated explants [35]. Whether other exogenously applied synthetic auxins, such as NAA and picloram, act similarly has not been studied because they are rarely used to induce this process in coniferous species [9,38,39]. However, these types of auxin may also promote the induction of SE in some coniferous tree species [15,38,40]. Combined application of picloram and BA to the medium results in high frequency of *Larix kaempferi* SE induction (62.8%) from mature zygotic embryos [15]. Additionally, the incorporation of NAA together with 2,4-D and BA in the medium improved the induction of embryogenic callus from apical dome sections of *Pinus kesiya* [38]. Despite numerous studies, the problem of the induction of somatic embryogenesis from different types of explants in conifers remains unsolved [41,42]. Therefore, knowledge and understanding of the mechanisms of SE induction with other auxins than 2,4-D would be highly desirable because auxins play a key role in this process. Our results may be a starting point for undertaking this type of research in this group of plants.

Ensuring that a large number of ET genotypes are capable of producing somatic embryos determines whether somatic embryogenesis can be practically applied [43]. The loss of the ability to proliferate in induced tissues and the consequent narrowing of the genetic diversity of the obtained embryogenic lines is one of the problems associated with the efficiency of this micropropagation method. This phenomenon has been documented in various coniferous species [44,45,46,47,48,49,50]. For example, only 5%–15% of the induced *P. abies* ET lines continued to grow during further cultures. Webb et al. [50] reported a 25%–50% capability in *P. glauca* ET lines to proliferate for longer than a few months. For *P. omorika*, of 127 induced ET lines, only 10 (7.87%) were able to proliferate for a longer time [49]. In turn, Kim and Moon [46] obtained only two cell lines capable of proliferation out of 52 lines induced from 11,388 explants. In our studies, most of the induced ET lines of both spruce species (approx. 2/3) also stopped proliferating or died, especially during the first 10 passages, regardless of the auxin used for tissue induction and proliferation. Only up to 30% of the ET lines of *P. abies* and up to 17% the ET lines of *P. omorika* obtained from each auxin treatment showed a stable ability to proliferate for a longer time. Our results reveal that this phenomenon was not affected by the auxin type used during ET maintenance in either spruce species. Since the decreased ratio of explants with proliferating ET to ET initiated from explants is a common reaction in coniferous species, some researchers proposed that the induction of stable ET lines should be the proper criterion for the assessment of SE induction frequency [51,52].

### 3.2. Effect of Auxin Treatment on the Physiological Condition of ET Lines and the Levels of Oxidative Stress and Guaiacol Peroxidase Activity

We noticed that ETs of both *Picea* sp. proliferated the most in the presence of NAA, although the obtained results were statistically significant only for *P. abies*. At the same time, the lowest ET growth was noticed in the presence of 2,4-D, an auxin usually added to the proliferation medium for conifer species. Moreover, in both spruce species, the production of H_2_O_2_ was the highest when 2,4-D was applied to the medium, although the obtained differences were statistically significant only in the case of the *P. abies* ET lines. This means that *P. abies* ETs are much more sensitive to the various auxin types added to the maintenance medium than the *P. omorika* ETs. It can suggest that the level of oxidative stress limited the growth of the *P. abies* cultures. A similar tendency was observed in *P. omorika* ET lines incubated in the presence of 2,4-D. The activity of the antioxidant system was also more differentiated in *P. abies* ETs and was dependent on the auxin type used. 2,4-D and picloram stimulated POX activity to the greatest extent in this spruce species. In turn, in *P. omorika*, POX activity was almost at the same level irrespective of the auxin type used, as found in our previous studies carried out on the representative ET lines of both tested spruce species [34]. In this research, guaiacol peroxidase activity was detected in explants (mature zygotic embryos) exposed to the chosen plant growth regulator systems, in 8-week-old embryogenic and nonembryogenic calluses, and the obtained embryogenic tissues [33]. The same pattern of guaiacol peroxidase activity was found in both tested spruce species, with the lowest level in explants and the highest level in 8-week-old calluses. A moderate level was detected in the induced and proliferated embryogenic tissues, regardless of the composition of the growth regulators added to the proliferation medium. The activity of guaiacol peroxidase was not dependent on the type or concentration of PGRs used during both the induction and proliferation of embryogenic cultures, although its activity increased in calluses treated with 2.4-D. This clearly indicates the participation of guaiacol peroxidase in somatic embryogenesis, and point POX activity as a biochemical marker of this process in both tested spruce species. In the current studies, the ETs of both spruces that proliferated the most in the presence of NAA had the lowest POX activity. This result means that the oxidative stress caused by the presence of NAA stimulates the growth of the ETs, especially in the case of *P. abies*, where the obtained results were statistically significant.

The majority of conifer species ETs are usually proliferated/maintained on the same composition medium as their induction medium in the presence of both auxins and cytokinins [52,53,54,55]. Sometimes a slight modification of the PGR content from induction to proliferation is performed [16,56], however, other medium modifications are necessary to improve ET growth and maintain their embryogenic potential [57]. For example, the proliferation medium may be supplemented with casein hydrolysate or L-glutamine as a source of organic nitrogen, or the sucrose or BA concentration may be decreased [53]. In our study, we reduced the BA dose by half (2.2 µM), which was combined with 9 µM auxins in the proliferation medium.

H_2_O_2_, as one of the forms of ROS, is produced via superoxidase during electron transport processes such as photosynthesis and respiration [22]. It is also induced during the exposure of plants to various abiotic and biotic stimuli, including extreme temperatures, UV radiation, phytohormones, dehydration, wounding [58]. Under stressful conditions, including in vitro cultures, H_2_O_2_ and other ROS may be produced in excess and accumulate in vitro, leading to oxidative stress [59]. The oxidative stress resulting from the imbalance between the production of ROS and the activity of the antioxidant system is closely related to plant in vitro cultures [8]. H_2_O_2_ is the most stable ROS molecule as it diffuses through the cell membranes and functions as a signalling molecule. It also plays an important role in the transduction of the defence signal in plants and can induce gene expression [60,61] and protein synthesis, leading to somatic embryogenesis in some plant species [62,63]. For example, H_2_O_2_ may induce the expression of genes encoding APX in germinating rice embryos [64] as well as the expression of genes encoding a catalase in the embryos and leaves of maize after wounding [65]. The clear correlation between treatment with H_2_O_2_ and the induction of somatic embryogenesis was demonstrated by Kairong et al. [63] in *Lycium barbarum*. The authors demonstrated that a few proteins (35~55 kDA with a isoelectric point (pI) level of 5~6) were synthesized by embryogenic callus after the treatment of the calluses with exogenously applied H_2_O_2_, suggesting that it induced gene expression at the mRNA level. In turn, Vranová et al. [61] demonstrated that the application of H_2_O_2_ to the medium may support cell division during the SE process. Somatic embryogenesis is a special cell differentiation process that is closely related to ROS metabolism [63], and many experiments have been performed to explain the role of oxidative stress in this process in several plant species [30,63,66,67,68,69]. In the case of conifers, such studies have also been undertaken [22,70,71,72], however, knowledge in this area is still limited and should be expanded.

### 3.3. Somatic Embryo Production

Our experiment showed that the type of synthetic auxin used during the proliferation stage of SE can affect the morphological structure of ETs and the ratio of some proembryonic structures in both spruce species. In turn, the influence of the tested auxins on the growth and development of somatic embryos was not statistically significant.

The proliferated ETs of conifers contain early stage somatic embryos (proembryos), which are represented by three characteristic proembryonic structures (PEM I, PEM II, and PEM III) according to Filonova et al. [18]. These PEMs differed in morphological structure and size. Proembryos are capable of regular division and multiplication, however, their further development into mature somatic embryos is inhibited in the presence of the PGRs initially added to the medium. Due to the lack of synchronization in the development of early-stage somatic embryos, the number of proembryos that successfully transition from the proliferation stage to the maturation stage is seriously limited. Consequently, only the most developed embryos (PEM III) respond to the maturation treatment, while many immature embryos die during this phase [73]. Typical proembryos at various developmental stages were also observed in our studies during the maintenance of the ET lines of both spruce species on media supplemented with various auxin types. However, we found that the type of synthetic auxin used affected the mean number of proembryos. It was evident that NAA promoted, to the greatest extent of all tested auxins, the intensity of the metabolic changes and of cell division, which resulted in a statistically significantly higher number of regenerated proembryogenic structures in ETs treated with NAA. Moreover, a pronounced effect of the auxin type was also noted in relation to the ratio and the growth of some proembryonic structures in both the *P. abies* and *P. omorika* ET lines. In the case of *P. abies*, these factors were promoted by 2,4-D, while in *P. omorika*, they were promoted by picloram. Our studies demonstrated that the tested auxins can affect not only the number and morphology of proembryos present in ETs during proliferation but also the level of synchronization of their development. This finding confirms that of Stasolla and Yeung [74] that manipulating the PGRs before transferring the ETs onto the maturation medium may increase the quality of the somatic embryos through, for example, the improvement of the organization of their PEM embryogenic regions. In conifer embryogenic cultures, the positive effects of liquid media on the synchronization of embryo development were reported during both the proliferation and maturation stages [73,75]. This is because the liquid culture medium provides better access to the medium components, stimulating a higher level of synchronization. Therefore, much more attention should be paid to testing various auxin types to improve early somatic embryo quality, particularly in liquid cultures. However, we must be aware that synchronization is dependent not only on culture conditions but also on the inherent capacity of ETs to generate more- or less-developed proembryos [73].

High exogenous auxin levels in the maintenance medium interfere with the polar auxin gradient that is established during embryogenesis and prevent correct apical-basal embryo patterning [76,77,78]. Therefore, to enable further somatic embryo development in conifers, the PGRs initially added to the induction and proliferation media are replaced with mild auxin (indole-3-butyric acid (IBA)), abscisic acid (ABA), and polyethylene glycol (PEG) [74,79,80,81]. The elimination of auxins and cytokinins is commonly used to improve the quality of somatic embryos in conifers [82]. For most coniferous species, mature somatic embryos are obtained from tissues routinely proliferated in the presence of 2,4-D and BA [19,83,84]. A few studies have shown that TEs proliferating before the maturation stage on NAA-supplemented media are able to regenerate somatic embryos in some conifers [16,85]. Li et al. [16] obtained mature somatic embryos after the prior treatment of *P. koraiensis* with NAA at 8.06 µM, BA at 1.1 µM, and kinetin at 1.16 µM. In turn, Hu et al. [85] demonstrated the possibility of obtaining mature somatic embryos of *Cunninghamia lanceolata* after using a low NAA concentration (1.08 µM) that was eight-times lower than that of the cytokinins used. Our previous studies revealed the possibility of generating mature somatic embryos of *P. abies* and *P. omorika* after the maintenance of ETs on a medium supplemented with picloram (9 µM) and BA (4.5 µM) [80]. Comparing the effects of 2,4-D, NAA and picloram used at the proliferation stage on the maturation stage in this study, we did not observe a statistically significant impact on the number of produced SESs or embryos at the cotyledonary stage in either spruce species. However, we noted a decrease in the mean number of somatic embryos in the NAA and picloram ET lines in *P. omorika*. The decrease may be connected with the concentrations of these PGRs in the proliferation medium being too high and consequently with the presence of residual amounts of these auxins in the maintained plant material. According to data from the literature, the PGR concentration in the culture medium before maturation determines the subsequent production of somatic embryos in coniferous species [86,87]. Klimaszewka et al. [86] observed the lowest number of mature somatic embryos of *Pinus strobus* when 2,4-D and BA were used at the highest concentrations (9 and 4.5 µM, respectively). The authors noted the possibility of the inhibition of the development of some somatic embryos on the maturation medium due solely to the residual amounts of PGRs in the embryogenic tissue.

### 3.4. Germination and Acclimatization

In this experiment, approximately 30% and only 5% of *P. abies* and *P. omorika* embryos, respectively, were able to develop into seedlings. Although more than approximately 95% of somatic embryos of both spruce species were able to germinate, most of them were not able to reach the next step of development. The highest percentage of somatic seedlings that were suitable for acclimatization (approximately 42%) was obtained in *P. abies* from the NAA ET lines, in which the highest level of hypocotyl and radicle development synchronization was also found. In turn, in the *P. omorika* NAA ET lines, hypocotyl and radicle development was the least synchronized of that in all lines. For comparison, somatic embryos of *Picea koraiensis* obtained from ET lines that were proliferated in the presence of NAA also developed poorly into seedlings [16]. According to Hay and Charest [88], the presence of functional shoots and roots testifies to the conversion of somatic embryos into somatic seedlings. The majority of the germinated *P. omorika* somatic embryos produced only thickened brown radicles, whose growth was limited regardless of the auxin type used. Therefore, only 12 germinated embryos of this spruce species were suitable for acclimatization.

In our studies, we routinely removed the exogenously applied auxins and cytokinin, and we used a medium with activated charcoal to eliminate the PGRs from the ETs before their transfer to the maturation media. Previous studies showed that transferring ETs proliferated in the presence of 2,4-D to a medium containing charcoal significantly reduced the concentration of this auxin in tissue cultures [89,90]. We assume that activated charcoal operates in a similar manner to NAA and picloram. However, although the tested auxins did not significantly affect embryo maturation, we observed their significant effect on further hypocotyl and radicle development in both spruce species during the germination stage. In *P. abies*, somatic embryos obtained from media supplemented with 2,4-D or picloram developed significantly longer hypocotyls and shorter radicles than the embryos obtained from NAA medium. Thus, the synchronization ratio was the best in germinated embryos obtained using NAA. In *P. omorika*, the opposite results were acquired. It seems that the exogenously applied auxins tested in this study caused slightly different metabolic changes in embryogenic cells that resulted in differences in physiological responses during the germination of the somatic embryos. In conifers, SE can be regulated by exogenously applied plant growth regulators that, together with endogenous phytohormones, control both the differentiation of cells and tissues and the establishment of structures and organs [91]. Therefore, to better understand the different effects of various exogenously applied auxins on somatic embryo germination in *P. abies* and *P. omorika*, a detailed study of hormonal changes during the proliferation and maturation stages should be performed in the future.

Many developmental processes in plants, including somatic embryogenesis, are regulated at the level of auxin biosynthesis, transport, perception and signalling [92]. Auxin together with cytokinin controls cell specification during the embryogenesis process [93]. It is connected with auxin polar transport, which plays a crucial role in apical-basal axis formation. During this process, auxin gradients mediated by the PIN-FORMED (PIN) protein family are established within the embryo before root and cotyledon formation [94]. Moreover, auxin-cytokinin cross-talk controls both shoot and root meristem development and the formation of lateral roots. In turn, auxin signalling through receptors and downstream signalling components may regulate the mechanisms responsible for organogenesis, embryogenesis, cell polarity establishment and vascular tissue development [92]. 

The problems with germination and plant conversion are still unresolved in many plant species [95]. To facilitate the proper development of embryos and their subsequent conversion into plants, the most commonly used auxin in the induction of somatic embryogenesis (2,4-D) is eliminated from the development and maturation steps [95,96]. It was reported that 2,4-D can cause changes at the genetic and epigenetic levels in cells [97,98] and consequently block the normal development of embryos [99,100]. It was documented in carrot cultures that the accumulation of 2,4-D inside the tissue, despite the treatment of embryogenic masses with an auxin-free medium, can arrest the further development of somatic embryos [95,101]. Therefore, to eliminate these problems, much more information should be obtained about the impact of a wider range of exogenously applied auxins on the metabolism of embryogenic tissues in the initial stages of somatic embryogenesis based on the currently available methods of biological and molecular analysis.

## 4. Materials and Methods

### 4.1. Plant Material

The embryogenic tissue cultures of *P. abies* and *P. omorika* were initiated from mature zygotic embryos. The explants were excised from seeds that were collected from trees growing in the ‘Zwierzyniec’ Experimental Forest near Kórnik (provenance Serwy) and in the Kórnik Arboretum (52°15′ N, 17°04′ E) in October 2017. The seeds were stored at 4 °C for 1–2 months before being used for ET initiation of ETs.

### 4.2. Initiation and Maintenance of Embryogenic Cultures

Explants were incubated on ½ Litvay et al. (1985) medium (LM) (½ of full concentration of media recommended by Litvay et al. [102]. The effects of three combinations of synthetic auxins and cytokinin on ET initiation were tested with 2,4-D, NAA or picloram at 9 µM and BA at 4.5 µM. The medium was supplemented with 10 g/L sucrose and 450 mg/L L-glutamine and solidified with Phytagel (5 g/L). The pH of the medium was stabilized at 5.8 before autoclaving. One hundred zygotic embryos (5 pieces per Petri dish) were used in each treatment variant. The experiment was repeated 3 times. The explants were incubated in darkness at 22 ± 1 °C. After 8 weeks of incubation, the ET initiation frequency was evaluated. The obtained and successfully proliferating ETs of both spruce species were maintained for over one year (14 months) under the same conditions and on the same medium but with a decreased concentration of BA (2.2 µM). In this paper, the obtained ET lines were labelled as the 2,4-D ET line, the NAA ET line and the picloram ET line according to the PGR variants used in the initiation media.

### 4.3. Effects of Auxin Treatment on the Physiological Condition of the ET Lines and the Levels of Oxidative Stress and Guaiacol Peroxidase Activity

After one year, the growth intensity as well as the oxidative stress level indicated by the H_2_O_2_ content and the guaiacol peroxidase activity of all ET lines that were able to continue to proliferate were analysed. The average weight gain was determined after 10 days of cultivation on the individual proliferation media. In total, 13 ET lines of *P. abies* (5 proliferated on 2,4-D, 3 on NAA and 5 on picloram) and 15 ET lines of *P. omorika* (5 proliferated on 2,4-D, 7 on NAA and 4 on picloram) were analysed. Three clumps per Petri dish in two to five repetitions depending on the species and the ET line were incubated to describe the ET proliferation intensity. For measuring oxidative stress, three technical repetitions from each ET line were used.

The H_2_O_2_ content was determined using the ferrithiocyanate method described by Sagisaka [103]. Samples were ground to a fine powder in liquid nitrogen and homogenized with 5 mL of 5% (*w*/*v*) trichloroacetic acid (TCA) containing 10 mM ethylenedinitrilotetraacetic acid (EDTA). Next, the homogenate was centrifuged at 20,000× *g* and 4 °C for 20 min. The total volume of the supernatant was analysed for H_2_O_2_ content.

Guaiacol peroxidase (POX) activity was measured as described by Chance and Maehly [104]. The samples (0.2 g each) were ground in liquid nitrogen and homogenized in 50 mM sodium phosphate buffer, pH 7.0, containing 0.2 mM EDTA and 20% polyvinylpolypyrrolidone (PVPP). The homogenates were centrifuged at 4 °C at 20,000× g for 20 min. Finally, the supernatants were analysed. The guaiacol peroxidase activity was measured spectrophotometrically in a guaiacol oxidation reaction at 470 nm for 1 min at 20 °C (using an extinction coefficient of ɛ = 26.6 mM/cm). The results correspond to the means ± SD of the values obtained from three different extracts and six measurements per extract. The protein content of crude enzyme extracts was estimated according to Bradford [105] using bovine serum albumin (BSA) as the standard.

### 4.4. Somatic Embryo Production

In both species, the effect of the tested auxin on somatic embryo production was described by the mean number of proembryos produced by each ET line. The presence of proembryos was detected with acetocarmine (2%) staining and observations under a light microscope (Axioskop 20, Carl Zeiss, New York, NY, USA). Fifteen microscopic preparations were analysed per ET line, further ten fields of vision were analysed within each preparation and mean number of proembryos was calculated on the base of obtained data. The size of the embryogenic region of each proembryo was measured with the AxioVisionL rel. 4.8 program, New York, NY, USA using a 5× lens. Proembryos with embryonic regions smaller than 63 µm were classified as PEM I; those with embryonic regions from 64 to 128 µm were classified as PEM II; and those with embryonic regions 128 µm or larger were classified as PEM III. These classifications were based on the measurement of the size of the embryogenic region of each proembryo at its base and correspond to the PEM I, PEM II, and PEM III proembryogenic structures according to Filonova et al. [18]. Then, the influence of auxins on the mean number (ratio) of proembryogenic structures and the size of their embryogenic regions was assessed.

To verify their capacity for somatic embryo regeneration (embryogenic potential), cell lines of *P. abies* and *P. omorika* were incubated for one week on ½ LM medium with the addition of activated charcoal (10 g/L). Next, the tested ETs were transferred to ½ LM maturation medium supplemented with 20 µM abscisic acid (ABA), 1 µM indole-3-butyric acid (IBA), 5% polyethylene glycol (PEG) 4000 and 34 g/L sucrose for 5 weeks. Three to five ET clumps (one per Petri dish) were examined depending on the spruce species and the ET line. The cultures were grown in a culture room at 22 ± 1°C with a light intensity of 35 µM/m^2^s^2^ and a 16/8 h (day/night) photoperiod. After this maturation period, the mean number of regenerated somatic embryos and the number of cotyledonary stage somatic embryos per gram of fresh weight of ET were assessed.

### 4.5. Germination and Acclimatization

Somatic embryos at the cotyledonary stage (approximately 1 mm in length) of 11 and 7 ET lines of *P. abies* and *P. omorika*, respectively, obtained from ETs growing in the presence of various auxins were cultured on ½ LM germination medium without PGRs and supplemented with sucrose at 10 g/L. Ten cotyledonary somatic embryos were placed in each Petri dish. In the end, 1129 (*P. abies*) and 246 (*P. omorika*) cotyledonary somatic embryos were selected for the germination stage. The number of somatic embryos tested depended on the ET line. The cultures were kept for 2 weeks in darkness and for the next 2 weeks in the light at an intensity of 35 µM/m^2^s^2^ with a 16/8 h (day/night) photoperiod at 22 ± 1 °C. The germination capacity of the tested embryos was assessed after 2 and 4 weeks of culture based on hypocotyl and radicle length measurements. Next, the germinated embryos were cultured on the same medium for an additional month to allow further development of both plant organs. Properly developed germinated embryos with hypocotyl lengths over 10 mm and radicle lengths over 5 mm (somatic seedlings) were selected and transferred to a substrate composed of a mixture of perlite and peat (1:3) to acclimatize them to the ex vitro conditions. Somatic plants of 11 ET lines of *P. abies* and 2 ET lines of *P. omorika* were transferred to the soil. The seedlings were planted under decreased light intensity in special containers that could regulate the air humidity.

### 4.6. Statistical Analysis

Data concerning ET initiation, the number of passages that the ETs survived, the efficiency of SESs maturation, and acclimatization were analysed using Pearson’s chi-square test (*p* < 0.05) and was used for assessing significance of ratio data. One-way analysis of variance (ANOVA) followed by the Tukey test was used to test the effect of auxin treatment on guaiacol peroxidase activity, H_2_O_2_ levels and parameters describing proembryo morphology, whereas data describing proembryo morphology and SESs germination were analysed with ANOVA followed by the Tukey–Kramer HSD test. Mean size of embryonic region of PEM III was calculated per each of preparation per ET line on the base of data collected from inspections of 10 fields of view, and those means were used for statistical analysis. Means were described as significantly different at *p* < 0.05, and all values associated with statistical tests used were presented. Data were presented as the mean values and standard errors. All analyses were performed with JMP 15 (SAS Institute Inc., North Carolina, NC, USA).

## 5. Conclusions

We demonstrated that 2,4-D, NAA and picloram can be applied to induce somatic embryogenesis in *P. abies* and *P. omorika*, however, the sensitivity of both *Picea* species varied in the different stages of this process. The presence of NAA in the first steps of somatic embryogenesis decreased oxidative stress and thus promoted the growth of ETs and enhanced the regeneration of proembryogenic structures.

In *P. omorika* ETs, the application of NAA and picloram decreased the production and maturation of somatic embryos, which could be related to the presence of residual amounts of NAA and picloram in the tissues. During the germination step, NAA significantly improved the synchronization of *P. abies* hypocotyl and radicle development to the greatest extent, resulting in the highest conversion of somatic embryos into seedlings of all treatments. Thus, our studies indicate that NAA may in some cases supports somatic embryo development in those spruce species better than 2,4-D, which is commonly used. We expect that the differences in the action of the tested auxins at individual stages of somatic embryogenesis and between spruce species result from their specific impact on the activity of the proteins associated with this process. We hope that our study will improve our knowledge about the regulation of the somatic embryogenesis process in these conifer species and will help to improve the efficiency of SE production in the future.

## Figures and Tables

**Table 1:** Embryogenic tissue initiation from mature zygotic embryos in *Picea abies* (L.) H. Karst and *P. omorika* (Pančić) Purk.

Species	PGR Type	Total Number of Lines (100%)	Number of Explants with ET Initiation (%)
*Picea abies*	2,4-D	232	17 (7.33%)
	NAA	249	19 (7.63%)
	picloram	229	24 (10.48%)
*Picea omorika*	2,4-D	250	55 (22.00%)
	NAA	271	44 (16.24%)
	picloram	264	40 (15.15%)

**Table 2:** Number (ratio) of the embryogenic lines of *Picea abies* and *P. omorika* in relationship to the plant growth regulators (PGR) type used during the induction of the embryogenic tissue (ET) lines and longevity of the obtained lines expressed as the number of passages.

Species	PGRs Type	Number of ET Lines According to Number of the Passages (%)			
		≤10	>10	Up to 40	Total (100%)
*Pic* *ea abies*	2,4-D	12 (70.59%)	5 (29.41%)	5 (29.41%)	17
	NAA	16 (84.21%)	3 (15.79%)	3 (15.79%)	19
	picloram	19 (79.17%)	5 (20.83%)	5 (20.83%)	24
*Picea omorika*	2,4-D	48 (87.27%)	7 (12.73%)	5 (9.09%)	55
	NAA	34 (82.93%)	7 (17.07%)	7 (17.07%)	41
	picloram	29 (76.32%)	9 (23.68%)	4 (10.53%)	38

**Table 3:** The growth intensity, level of H_2_O_2_ and guaiacol peroxidase (POX) activity in embryogenic lines of *Picea abies* and *P. omorika* in relationship to the PGR type used during the induction of the ET lines. (mean ± standard error). Different letters within columns indicate statistically significant differences between means for PGRs type within species (*p* < 0.05).

Species	PGRs Type	ET Growth [g]	H_2_O_2_ [nmol/gFW]	POX [mM/gFW]
*Picea abies*	2,4-D	0.58 ± 0.1 b	2.60 ± 0.2 a	54.92 ± 5.8 ab
	NAA	1.06 ± 0.1 a	2.25 ± 0.2 ab	34.76 ± 7.5 b
	picloram	0.78 ± 0.1 ab	2.01 ± 0.12 b	64.41 ± 5.8 a
*Picea omorika*	2,4-D	0.90 ± 0.2 a	2.01 ± 0.2 a	32.97 ± 4.9 a
	NAA	1.21 ± 0.1 a	1.61 ± 0.1 a	28.78 ± 4.2 a
	picloram	1.06 ± 0.2 a	1.46 ± 0.2 a	33.26 ± 5.5 a

**Table 4:** The number (ratio) of proembryogenic structures and the mean size of the embryogenic region (µm, mean ± standard error). Different letters within columns indicate statistically significant differences between means for PGRs type within species (*p* < 0.05).

Species	PGRs Type	PEM I	PEM II	PEM III
**Number (Ratio) of Proembryogenic Structures**				
*Picea abies*	2,4-D	125 (16.82%)	309 (41.59%) b	309 (41.59%) a
	NAA	129 (15.07%)	445 (51.99%) a	282 (32.94%) b
	picloram	160 (15.53%)	440 (42.72%) b	430 (41.75%) a
*Picea omorika*	2,4-D	592 (22.21%) ab	1345 (50.47%) a	728 (27.32%) b
	NAA	1353 (28.67%) a	2352 (49.83%) a	1015 (21.50%) c
	picloram	251 (19.43%) b	597 (46.21%) b	444 (34.37%) a
**Size of the Embryogenic Region (µm, Mean ± SEM)**				
*Picea abies*	2,4-D	48.71 ± 1.3	95.50 ± 1.3	212.62 ± 4.4 a
	NAA	49.99 ± 1.4	95.73 ± 1.5	179.30 ± 3.5 b
	picloram	50.18 ± 1.3	95.04 ± 1.2	205.45 ± 3.5 a
*Picea omorika*	2,4-D	48.49 ± 0.6	91.81 ± 0.7 b	183.24 ± 3.3
	NAA	48.55 ± 0.5	91.17 ± 0.6 b	179.69 ± 2.8
	picloram	49.28 ± 0.7	94.47 ± 0.8 a	190.89 ± 4.3

**Table 5:** Hypocotyl and radicle length of somatic embryos after two weeks of growth in the darkness and hypocotyl and radicle length and ratio of hypocotyl length to radicle length after two weeks of growth in light of germinated *P. abies* and *P. omorika* somatic embryos in relationship to the PGR type used during the induction of the ET lines. Different letters within columns indicate statistically significant differences between means for PGRs type within species (*p* < 0.05).

Species	PGRs Type	SE after Two Weeks in the Darkness		SE after Two Weeks on the Light		
		Hypocotyl Length (mm)	Radicle Length (mm)	Hypocotyl Length (mm)	Radicle Length (mm)	HL/RL Ratio
*Picea abies*	2,4-D	9.91 ± 0.4 a	2.76 ± 0.1 a	11.08 ± 0.4 a	3.75 ± 0.2 b	1.81 ± 0.1 a
	NAA	6.94 ± 0.3 c	2.17 ± 0.1 b	8.61 ± 0.3 b	5.32 ± 0.4 a	1.51 ± 0.1 b
	picloram	8.46 ± 0.2 b	2.71 ± 0.1 a	10.37 ± 0.2 a	4.53 ± 0.2 ab	1.77 ± 0.1 a
*Picea omorika*	2,4-D	3.79 ± 0.2 b	2.52 ± 0.1 a	4.98 ± 0.2 ab	2.97 ± 0.1 a	1.96 ± 0.4 b
	NAA	4.88 ± 0.3 a	1.62 ± 0.1 b	5.98 ± 0.4 a	1.64 ± 0.1 b	4.41 ± 0.4 a
	picloram	4.42 ± 0.5 ab	2.75 ± 0.3 a	4.92 ± 0.5 b	3.42 ± 0.5 a	1.78 ± 0.1 b

**Table 6:** Number of somatic embryos classified as suitable for acclimatization obtained from embryogenic lines of *Picea abies* and *P. omorika* in relationship to the PGR type used during the induction of the ET lines.

Species	PGRs Type	Number of SE	
		Germinated in Vitro	Suitable for Acclimatization
*Picea abies*	2,4-D	234	31
	NAA	296	123
	picloram	589	173
*Picea omo* *rika*	2,4-D	166	10
	NAA	58	0
	picloram	12	2

## Abbreviations

ABA	abscisic acid
BA	benzyladenine
BSA	bovine serum albumin
2,4-D	2,4-dichlorophenoxyacetic acid
EDTA	ethylenedinitrilotetraacetic acid
ET	embryogenic tissue
H_2_O_2_	hydrogen peroxide
LM	Litvay et al. (1985) medium
NAA	1-naphtaleneacetic acid
PEG	polyethylene glycol
PEM I	embryogenic stucture type I
PGR	plant growth regulator
Picloram	4-amino-3,5,6-trichloropicolinic acid
POX	guaiacol peroxidase
PVPP	polyvinylpolypyrrolidone
ROS	reactive oxygen species
SE	somatic embryogenesis
TCA	trichloroacetic acid
